# Long-term mental health outcomes after unintentional burns sustained during childhood: a retrospective cohort study

**DOI:** 10.1186/s41038-018-0134-z

**Published:** 2018-11-13

**Authors:** Janine M. Duke, Sean M. Randall, Thirthar P. Vetrichevvel, Sarah McGarry, James H. Boyd, Suzanne Rea, Fiona M. Wood

**Affiliations:** 1 Burn Injury Research Unit, Faculty Health and Medical Sciences, The University of Western Australia, M318 35 Stirling Highway, Crawley, Perth, Western Australia 6009 Australia; 2 Centre for Data Linkage, Curtin University, Perth, Western Australia Australia; 3 Curtin Medical School, Curtin University, Perth, Western Australia Australia; 4 School of Occupational Therapy Social work and Speech Pathology, Curtin University, Perth, Western Australia Australia; 5 Burns Service of Western Australia, Royal Perth Hospital and Princess Margaret Hospital, Perth, Western Australia Australia

**Keywords:** Paediatric burns, Depression, Anxiety, Substance abuse, Longitudinal study, Mental health

## Abstract

**Background:**

Burns are a devastating injury that can cause physical and psychological issues. Limited data exist on long-term mental health (MH) after unintentional burns sustained during childhood. This study assessed long-term MH admissions after paediatric burns.

**Methods:**

This retrospective cohort study included all children (< 18 years) hospitalised for a first burn (*n* = 11,967) in Western Australia, 1980–2012, and a frequency matched uninjured comparison cohort (*n* = 46,548). Linked hospital, MH and death data were examined. Multivariable negative binomial regression modelling was used to generate incidence rate ratios (IRR) and 95% confidence intervals (CI).

**Results:**

The burn cohort had a significantly higher adjusted rate of post-burn MH admissions compared to the uninjured cohort (IRR, 95% CI: 2.55, 2.07–3.15). Post-burn MH admission rates were twice as high for those younger than 5 years at index burn (IRR, 95% CI 2.06, 1.54–2.74), three times higher for those 5–9 years and 15–18 years (IRR, 95% CI: 3.21, 1.92–5.37 and 3.37, 2.13–5.33, respectively) and almost five times higher for those aged 10–14 (IRR, 95% CI: 4.90, 3.10–7.76), when compared with respective ages of uninjured children. The burn cohort had higher admission rates for mood and anxiety disorders (IRR, 95% CI: 2.79, 2.20–3.53), psychotic disorders (IRR, 95% CI: 2.82, 1.97–4.03) and mental and behavioural conditions relating to drug and alcohol abuse (IRR, 95% CI: 4.25, 3.39–5.32).

**Conclusions:**

Ongoing MH support is indicated for paediatric burn patients for a prolonged period after discharge to potentially prevent psychiatric morbidity and associated academic, social and psychological issues.

## Background

Burns are a common reason for paediatric emergency department visits and hospitalisations in the developed world, with the highest incidence occurring among those younger than 5 years of age [1–4]. Over the past decades with significant advances in treatment, health outcomes after burns have shifted from being measured in terms of in-hospital mortality to include functional rehabilitation and quality-of-life. Nonetheless, burns remain one of the most devastating injuries that can be sustained by a child. Surgical and medical treatments for the acute injury are both painful and stressful and burn scars are associated with social anxiety, avoidance and reduced quality-of-life [5, 6].

Evidence exists of increased levels of anxiety and depression amongst adults who sustain burns [7–12]; however, information regarding long-term effects on post-burn psychological health is more restricted. To date, mental health (MH) of paediatric burn patients has been an important focus of burns research. However, assessment of the psychiatric health of paediatric burn patients has often been limited by small sample sizes, relatively short post-burn follow-up time periods and lack of comparator or control groups [13–18]. Studies by Meyer et al. [14] (*n* = 101) and Goodhew et al. [19] (*n* = 227) reported on long-term psychiatric health of adult survivors of paediatric burns using self-report and structured clinical interviews; both studies identified high rates of current and lifetime psychopathology. Recently, we conducted a retrospective cohort study of all patients hospitalised with unintentional burns in Western Australia (2000–2012) and found significantly elevated admission rates for MH conditions for 5 years post-burn; however, rates were highest in those younger than 18 years of age at the time of burn (incidence rate ratios (IRR), 95% confidence intervals (CI) 6.28, 3.00–13.14), followed by those aged 18–60 years (IRR, 95% CI: 5.14, 3.59–7.35) and those over 60 years (IRR, 95% CI: 2.97, 1.38–6.39) [20].

This study used ‘whole-of-population’ record linkage of hospital morbidity, death and MH case registers to provide a more detailed assessment of the long-term MH outcomes of children hospitalised with unintentional burns and to compare with a cohort of age- and gender-matched uninjured children.

## Methods

This retrospective cohort study used population-based health data from the Western Australia Population-based Burn Injury Project (WAPBIP) and included linked records from the Western Australian Hospital Morbidity Data System (hospital records), death register and Mental Health Information System (MHIS). The MHIS is a comprehensive psychiatric case register of all contacts with in-patient MH services (private and public hospitals) in Western Australia since 1966. Records were linked and extracted by the Department of Health, Western Australia Data Linkage System (WADLS) [21]. Project approvals were granted by Human Research Ethics Committees of the Western Australian Department of Health and the University of Western Australia. Several papers have been published using WAPBIP data; methods including cohort selection and analyses have been previously published [22–25].

The burn cohort included all children hospitalised with an index (first) admission for an unintentional burn in Western Australia from 1 January 1980 to 30 June 2012. The population-based comparison uninjured cohort was randomly selected from the Western Australian Birth Registrations and excluded any person with a record of a traumatic injury admission during the study period. The uninjured comparison cohort was frequency-matched on birth year (± 2 years), gender and geographic region of the burns cases (ratio ~ 4:1) for each year from 1980 to 2012.

Data were linked to each cohort (burn, non-injury) for the period 1975–2012, including indices of geographic remoteness [26] and socio-economic disadvantage [27]. The socio-economic disadvantage index is derived from 40 items in the Australian census and is highly correlated with lifestyle risk factors (e.g. nutrition, physical activity, alcohol, smoking and substance abuse) [28–31]. Study variables included principal/additional diagnoses, age, gender, indigenous status, admission and discharge dates, residential postcode and census district, burn characteristics (percentage of total body surface area (burned) (%TBSA), burn depth, anatomical site) and external cause codes. Principal diagnosis data in the MHIS were used with International Classification of Diseases (ICD) Chapter 6 codes to identify and classify admissions for MH and drug and alcohol conditions. Principal external cause codes were used to identify admissions for self-harm. Mortality data included the date and cause of death.

%TBSA was classified as minor burns (TBSA < 20%), severe (TBSA ≥ 20%) and unspecified. A Charlson Comorbidity Index (CCI) [32] was generated for each child using diagnosis data with a 5-year lookback period [33] (classified CCI = 0; ≥ 1). Variables were generated to identify admissions during the 5-year lookback period for MH conditions, self-harm and drug/alcohol abuse. Socio-economic disadvantage was partitioned into quintiles (most to least disadvantaged), and geographic remoteness was classified as major cities, inner regional, outer regional, remote and very remote.

Individuals were followed up after burn hospital discharge until death or the end of the study period (30 June 2012). The total number of years a person was at risk (person-years) was estimated from the final discharge date (study index date) for the burn cases, and this date was used for the respective frequency-matched uninjured controls. The total number of annual admissions and summed length of stay (LOS) (days) for post-index MH conditions were used as outcome measures. Analyses were undertaken sub-cohorts defined by %TBSA and age group at study start (0–4, 5–9, 10–14, 15–17 years). Categorical variables were compared using *χ*^2^ tests; level of statistical significance was 0.05.

Adjusted IRR and 95% CI examining annual total MH admissions (combined incident admission and readmissions) were generated using multivariable negative binomial regression. Separate negative binomial models were analysed for those with and without a record of prior admission for MH conditions (identified during 5-year look-back period). Socio-demographic (gender, indigenous status, age, social disadvantage, remoteness of residence), year of admission and health status variables (baseline comorbidity, previous MH admission) were included as covariates in the models.

In addition, sub-groups of MH conditions by ICD categories were analysed (psychotic disorders F20-F29; combined mood and anxiety disorders F30-F48), as well as behavioural and MH conditions caused by drug and alcohol abuse (F10-F19). ICD9 codes were mapped to ICD10 codes. MH sub-group conditions were also analysed by age group at time of burn admission. Statistical analyses were performed using Stata version 12 (StataCorp. LP, College Station, USA).

## Results

### Cohort characteristics

There were 11,967 children (< 18 years) hospitalised for a first unintentional burn. Males represented 64% (*n* = 7583), and median age was 3 years (interquartile range (IQR) 1–10 years). With respect to TBSA, 1.4% (*n* = 164) ≥ 20% TBSA; 46% (*n* = 5487) < 20% TBSA; TBSA unspecified for 53% (*n* = 6257). Analysis of LOS suggests that those with unspecified TBSA (median LOS 4 days, IQR 1–10) were most likely < 20% TBSA (median LOS 4 days, IQR 1–9) compared to ≥ 20% TBSA (median LOS 24.5 days, IQR 7.5–45). This cohort excluded those with burns resulting from self-harm (*n* = 21) and assault (*n* = 38).

Burn site depth recorded 9% (*n* = 1100) full thickness, 44% (*n* = 5294) partial thickness, 15% (*n* = 1737) erythema (first degree) and 35% (*n* = 4131) unspecified burn thickness. Individuals could have multiple burn sites and depths recorded. Burn sites included head and neck (21%, *n* = 2441), trunk (26%, *n* = 3152), upper limbs/hands (40%, *n* = 4748), lower limbs/feet (34%, *n* = 4001), eye (4%, *n* = 492) and respiratory tract or other internal organs (2%, *n* = 203). In the burn cohort, 31% (*n* = 3714) had an admission for non-burn injury (before or after burn).

In the burn cohort, 12 (0.1%) died from their burn in hospital and 215 (2%) died before the end of the study period (22% (*n* = 47) were due to suicide). The non-injured cohort contained 46,548 individuals with no injury hospitalisation during the study period. By the end of the study period, 1% (*n* = 340) had died; 19% (*n* = 66) were due to suicide.

Table 1 contains a summary of socio-demographic and health care characteristics. In the burn cohort, 11 children had a previous admission for a stress adjustment disorder or other anxiety-related condition; another 11 children had admissions relating to depression with smaller number with admissions for eating disorders and psychotic conditions.

**Table 1:** Baseline demographic and pre-existing health status factors for burn and uninjured cohorts

Characteristics	Uninjured *N* (%)	Burn injury *N* (%)	*P* value
Total	46,548	11,967	
Demographic			
Gender			
Male	29,238 (62.8)	7583 (63.4)	0.090
Age			
0–4	27,061 (58.1)	6794 (56.8)	0.113
5–9	6966 (15.0)	1794 (15.0)	
10–14	6792 (14.6)	1826 (15.3)	
15–17	5729 (12.3)	1494 (12.5)	
Aboriginality			
Yes	2136 (4.6)	2196 (18.4)	< 0.001
Social disadvantage quintiles^a^			
Quintile 1 (most disadvantaged)	5826 (12.5)	2622 (21.9)	< 0.001
Quintile 2	11,740 (25.2)	3903 (32.6)	
Quintile 3	8394 (18.0)	2393 (20.0)	
Quintile 4	7860 (16.9)	1461 (12.2)	
Quintile 5 (least disadvantaged)	12,484 (26.8)	1410 (11.8)	
Remoteness^b^			
Major city	31,448 (67.6)	6184 (51.7)	< 0.001
Inner regional	4772 (10.3)	1262 (10.5)	
Outer regional	5259 (11.3)	1932 (16.1)	
Remote	2933 (6.3)	1379 (11.7)	
Very remote	1784 (3.8)	1076 (9.0)	
Health status			
Any comorbidity (CCI ≥ 1)^c^	526 (1.1)	263 (2.2)	< 0.001
Prior admission for mental health condition^d^	29 (0.1)	35 (0.3)	< 0.001
Prior admission for self-harm	14 (0.0)	24 (0.2)	< 0.001
Prior admission for drugs/alcohol	10 (0.0)	12 (0.1)	< 0.001
Congenital abnormality	1114 (2.4)	375 (3.1)	< 0.001

^a^Socio-Economic Index for Areas (SEIFA) socio-economic disadvantage quintiles; missing values 1.0% burn, 0.5% no injury

^b^Accessibility Remoteness Index of Australia, revised version (ARIA+) remoteness classification; missing values 0.6% burn, 0.8% no injury

^c^Comorbidity based on derived Charlson Comorbidity Index (CCI) using 5-year look-back period

^d^Principal diagnosis record of hospitalisation for mental health condition using 5-year look-back period

The median length of follow-up was 18 years (IQR 10–26 years) for both the burn and uninjured cohorts.

### Admission rates

In the burn cohort, 550 individuals had at least one post-burn admission for a MH condition for a total of 1773 hospital admissions. In the uninjured cohort, 682 individuals had a total of 2168 MH. In addition, there were a total of 972 post-burn admissions in the burn cohort for mental and behavioural conditions caused by drugs and alcohol (compared to 612 in the uninjured cohort); these occurred in 476 and 347 individuals in the burn and uninjured cohorts, respectively. In the burn cohort, 317 individuals recorded a total of 710 post-burn admissions for self-harm.

In the burn cohort, median LOS for MH conditions was 4 days (IQR 1–12 days) compared with 3 days (IQR 0–13 days) in the uninjured cohort, 1 day (IQR 0–4 days) for admissions for drugs/alcohol (vs. 1 day (IQR 0–5 days) in the uninjured cohort) and 1 day (IQR 1–3 days) for admissions for self-harm.

By the end of the study period, 4.6% of the burn cohort had been admitted for a MH condition, 4.0% for drugs/alcohol and 2.7% for self-harm. In the uninjured cohort, 1.5% had been admitted for a MH condition and 0.8% for drugs/alcohol. The median time to first admission for a MH condition was 6421 days in the burn cohort (minimum (min) 3 days, maximum (max) 11,740 days) and 6485 days in the uninjured cohort (min 4 days, max 11,436 days).

**Fig. 1:**
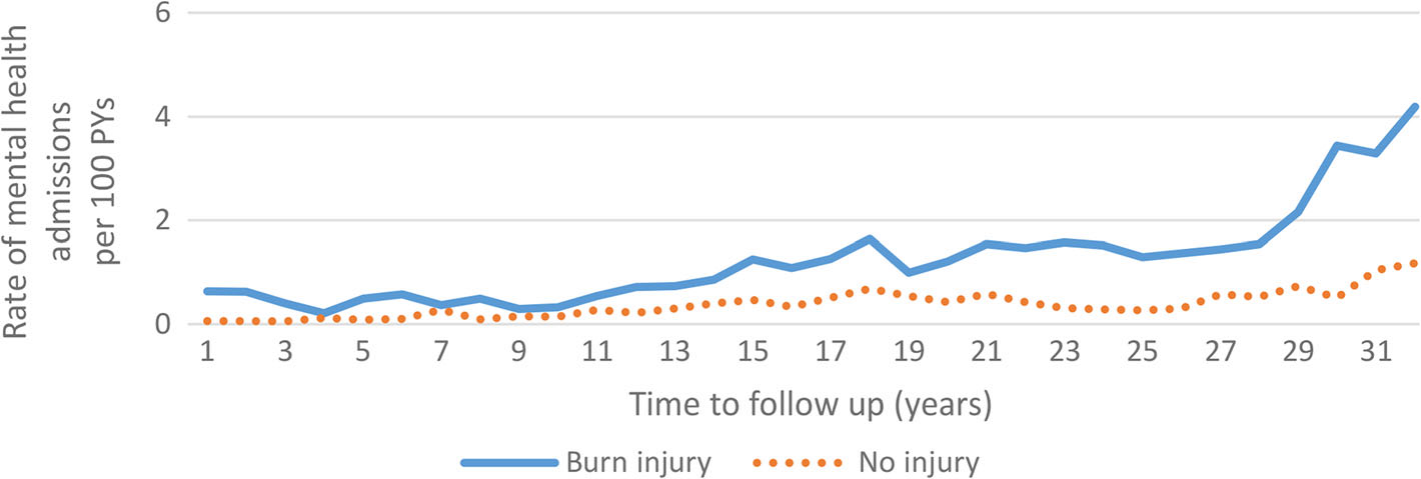
Unadjusted (observed) rates of mental health hospital admissions (per 100 person-years (PYs)) among those with burns versus no injury per year of follow-up post-burn (burn cohort) and index study start (uninjured cohort)

A breakdown of MH sub-conditions is found in Table 2. The burn cohort had a slightly higher proportion of anxiety-related disorders, with a particularly large proportion of admissions for stress/adjustment disorder. Crude (observed) rates of admissions for MH conditions are shown in Fig. 1. The burn cohort had higher annual admission rates for MH conditions compared to the uninjured cohort, with MH admissions increasing over time. The increases over time most likely reflect an age effect (e.g. older individuals have higher MH admission rates than children) and a cohort effect, whereby MH admissions were generally greater after 2000 compared with that observed during the 1980s.

**Table 2:** Number (%) of post-index admissions for mental health conditions for the burn and uninjured cohorts for the study period 1980–2012

	Burn injury *N* (%)	Uninjured *N* (%)
Mental health conditions		
Psychotic disorders	489 (27.6)	559 (25.8)
Schizophrenia	305 (17.2)	357 (16.5)
Schizoaffective disorders	26 (1.5)	55 (2.5)
Other psychotic disorders	158 (8.9)	147 (6.8)
Mood disorders	629 (35.5)	851 (39.3)
Major depressive disorder	493 (27.8)	597 (27.5)
Bipolar disorder/manic episode	95 (5.4)	221 (10.2)
Other mood disorders	41 (2.3)	33 (1.5)
Anxiety disorders	578 (32.6)	566 (26.1)
Anxiety disorder	77 (4.3)	177 (8.2)
Stress/adjustment disorder	442 (24.9)	315 (14.5)
Dissociative/conversion disorders	30 (1.7)	12 (0.6)
Other anxiety/stress disorders	29 (1.6)	62 (2.9)
Eating disorders	75 (4.2)	188 (8.7)
Other mental disorders	2 (0.0)	4 (0.2)
Total	1773 (100.0)	2168 (100.0)
Self-harm		
Intentional poisoning	499 (70.3)	311 (100.0)
Sharp object	120 (16.9)	0^a^
Other self-harm	91 (12.8)	0^a^
Total	710 (100.0)	311 (100.0)
Mental and behavioural disorders due to psychoactive substance use		
Alcohol-related disorders	506 (52.1)	252 (41.2)
Opioid-related disorders	52 (5.3)	84 (13.7)
Cannabis-related disorders	64 (6.6)	50 (8.2)
Stimulant-related disorders	128 (13.2)	115 (18.8)
Other psychoactive substance-related disorders	222 (22.8)	111 (18.1)
Total	972 (100.0)	612 (100.0)

^a^Individuals with an injury (including self-harm injury) were excluded from this uninjured cohort

The burn cohort had a significantly higher adjusted rate of MH admissions over the study period compared to the uninjured cohort (IRR, 95% CI: 2.55, 2.07–3.15). Refer to Table 3. The wide 95% CI associated with prior MH is related to small numbers. Analyses by age group found the rate of MH admissions to be twice as high (IRR, 95% CI: 2.06, 1.54–2.74) for those younger than 5 years at index burn, three times higher for those aged 5–9 and 15–18 (IRR, 95% CI: 3.21, 1.92–5.37 and 3.37, 2.13–5.33, respectively) and nearly five times higher for those aged 10–14 (IRR, 95% CI: 4.90, 3.10–7.76), when compared with respective uninjured age groups.

**Table 3:** Adjusted incidence rate ratios (IRR) and 95% confidence intervals (CI) for post-injury admissions for mental health conditions comparing those with burn injury with non-injured, Western Australia, 1980–2012

Characteristics	Adjusted IRR^d^	95% CI
Burn	2.55	2.07–3.15
Female	1.79	1.44–2.22
Indigenous (yes vs no)	2.77	2.07–3.70
Age at index event		
0–4 years (ref)	1.00	–
5–9 years	1.65	1.22–2.24
10–14 years	4.31	3.19–5.82
15–17 years	4.39	3.16–6.09
Social disadvantage^a^ (increasing advantage)	1.10	1.01–1.19
Remoteness^b^ (increasingly remote)	1.06	0.97–1.15
Year of index admission (increasing)	0.93	0.92–0.95
Previous mental health admission	56.30	22.00–144.05^d^
Prior self-harm admission	5.83	2.14–15.89^d^
Prior drug/alcohol admission	2.36	0.94–5.93^d^
Pre-existing comorbidities^c^	2.26	1.28–5.93

^a^Socio-Economic Index for Areas (SEIFA) socio-economic disadvantage

^b^Accessibility Remoteness Index of Australia, revised version (ARIA+) remoteness classification

^c^Comorbidity derived using Charlson Comorbidity Index (CCI) 5-year look-back period

^d^Wide 95% CI related to small numbers with prior admissions

Twofold higher rates of MH admissions were found across TBSA classifications, and rate of similar magnitude was found for those with burns to more visible sites of head and neck (IRR, 95% CI: 2.38, 1.67–3.40).

**Fig. 2:**
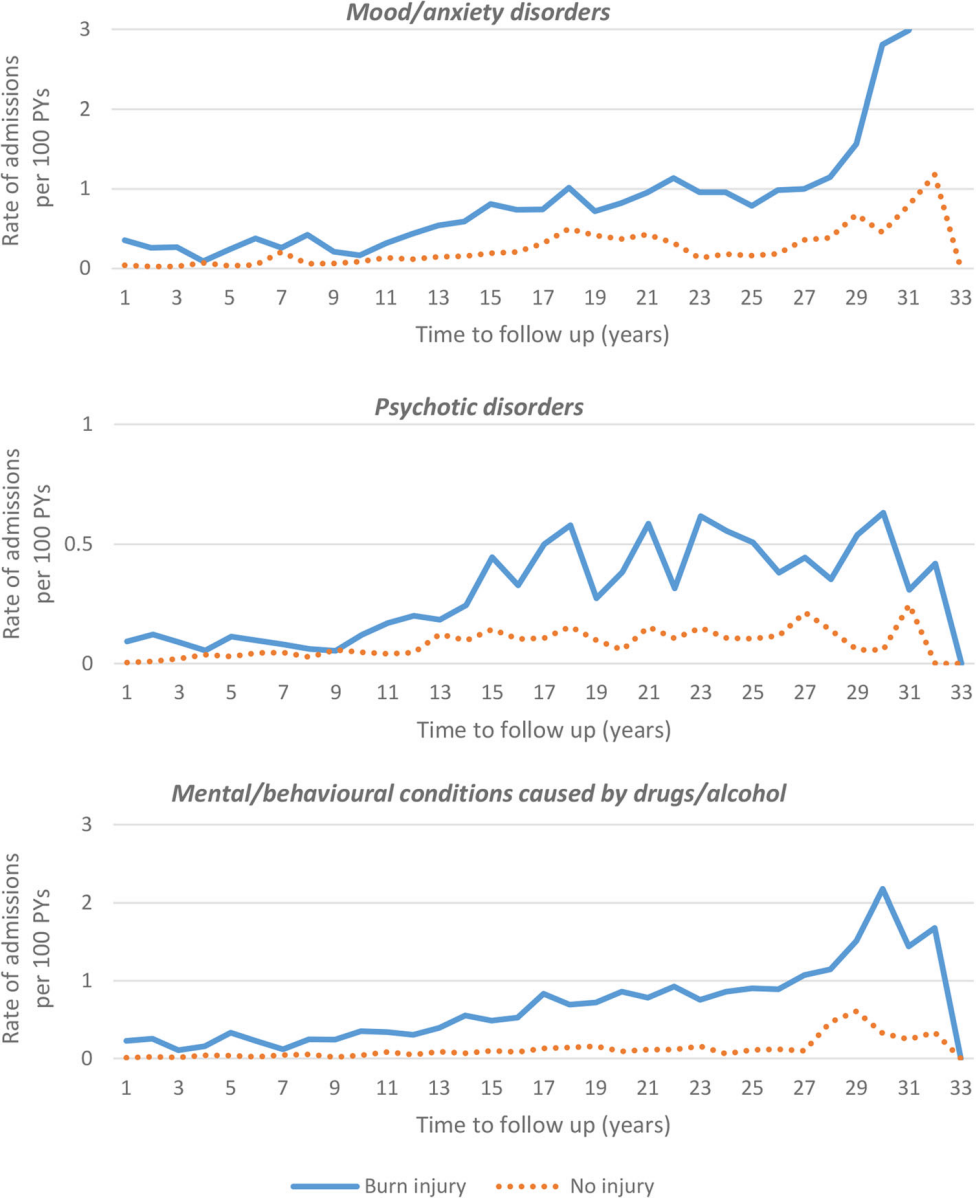
Unadjusted (observed) rates of hospital admissions (per 100 person-years (PYs)) by sub-groups (mood/anxiety disorders, psychotic disorder and mental/behavioural conditions caused by drug/alcohol) of mental health conditions among those with burns versus no injury per year of follow-up post-burn (burn cohort) and index study start (uninjured cohort)

Crude (observed) rates of hospitalisations for MH conditions by sub-group are shown in Fig. 2. After adjustment, the burn cohort had statistically significantly elevated admission rates when compared to the uninjured cohort for mood and anxiety disorders (IRR, 95% CI: 2.79, 2.20–3.53), psychotic disorders (IRR, 95% CI: 2.82, 1.97–4.03) and for mental and behavioural conditions relating to drug and alcohol abuse (IRR, 95% CI: 4.25, 3.39–5.32). Table 4 contains results of adjusted IRR for MH sub-group conditions by age at time of burn admission.

**Table 4:** Adjusted incidence rate ratios (IRR) and 95% confidence intervals (CI) for post-injury admissions for mental health conditions comparing those with burn injury with non-injured, by age group

Age at index event	Adjusted IRR (95% CI)^a^		
	Mood and anxiety disorders	Psychotic disorders	Drug and alcohol abuse
0–4 years	2.24 (1.68–2.97)	2.59 (1.56–4.29)	2.38 (1.74–3.26)
5–9 years	3.36 (1.81–6.23)	2.57 (1.23–5.35)	8.30 (4.62–14.90)
10–14 years	3.42 (2.24–5.21)	4.15 (2.13–8.10)	6.44 (4.43–9.37)
15–17 years	2.50 (1.50–4.17)	1.77 (0.79–3.96)	5.15 (3.25–8.16)

^a^Negative binomial regression models adjusted for gender, indigenous status, social disadvantage, geographic remoteness to services, prior mental health admissions, pre-existing comorbidity, admission year

## Discussion

This study found that the paediatric cohort with unintentional burns experienced elevated rates of post-burn admissions for a psychiatric condition at a rate 2.6 times higher than the comparison uninjured cohort. Examination of burn severity (TBSA) found post-burn MH admission rates were at least twice as high (adjusted IRR between 2.00 and 2.81) for both severe and minor classifications when compared with the uninjured. Those with burns to more visible anatomical sites (that is, face and head) also had similarly elevated admission rates (adjusted IRR 2.38).

While significantly elevated post-burn MH rates were found for all age groups, children between the age of 10 and 15 years at the burn admission experienced the highest admission rate at almost five times higher than that for uninjured children. While future research is required to determine causative factors for this increase, it could be suggested that these results reflect a global pattern whereby mental disorders and MH problems have increased considerably among adolescents in the past 20–30 years [34]. The rise has been driven by social change, including disruption of family structure, growing youth unemployment and increasing educational and vocational pressures [34]. Post-burn admissions for mood and anxiety disorders were increased for all age groups; however, those aged between 5 and 15 years at time of burn had an admission rate three times higher than uninjured children. Admissions for psychotic disorders were significantly higher for children younger than 15 years at index burn. Admission rates for substance and alcohol abuse were particularly concerning: eight times higher for children 5–9 years at time of burn, six times higher for 10–14 years and five times higher for those 15–17 years, compared with uninjured children.

As a consequence of surgical and rehabilitation interventions for burn scars over a long period of time, children can experience ongoing medical traumatic stress in addition to that associated with the initial trauma, which leads to behavioural and emotional changes [35]. Additionally, psychological reasons for the increased risk for MH problems can include self-esteem and body image issues. Research reports that burn survivors struggle with unwanted and often distressing social reactions all throughout their life [36–38]. A recent review of the psychological effects of childhood burns estimated that between 25 and 30% of preschool children experienced significant traumatic stress reactions during the first month post-burn [36]. For school-aged children, these rates have been reported to be a lot lower within the same time period, with rates reported to be 3–5% 3 months post-burn [39] and behavioural rates have been reported as normal in other studies [40]. However, prevalence rates of adverse psychological symptoms have been shown to increase over time with 10 to 20% of school-aged children reporting symptoms of post-traumatic stress disorder (PTSD) several years after the initial burn [41]. This suggests that the risk of developing adverse psychological symptoms may increase with growing older as well as increased time post-burn injury. Results from this current study highlight the discrepancy between short-term follow-up rates and the long-term higher levels of risk found in this cohort.

The impacts of the initial burn trauma, disfigurement and effects on one’s body image and associated social stigma among paediatric burn survivors, have been the focus of a number of studies [8, 14, 42–44]. Latency age and teen-age burn survivors appear to make adjustments to their core personal values by reducing and shifting the importance of physical appearance to emphasising other personal attributes [15]. However, transitioning from childhood to adulthood and more independent living introduces new challenges related to new personal contacts and relationships, and less familiar environments, which may cause individuals to re-evaluate their situations and self [15].

Burns are associated with acute and potentially persistent depression of humoral and cell-mediated immunity [45–47], sustained levels of oxidative stress [48, 49] and prolonged elevation of stress hormone levels [50, 51] with pathophysiological effects persisting for at least 3 years post-burn [52]. In addition, cytokines and neuropeptides, such as oxytocin, are related to pain and psychological distress [53, 54], and significant evidence suggests oxytocin has a neuromodulation role in (traumatic) stress and anxiety [53, 55]. Evidence demonstrates that such systemic responses may occur after both severe and minor burns [56–59]. Given that the majority of paediatric burn admissions are for minor burns, these findings have important health implications.

Research in the field of depression has identified inflammation and cell-mediated immune activation, and activation of the compensatory anti-inflammatory reflex system, as key factors [60–65] and that increased oxidative and nitrosative stress also accompanies depression [66]. Results of a meta-analysis study confirm that inflammatory, cell-mediated and negative immunoregulatory cytokines are found in psychiatric disorders such as mania and bipolar disorder [67]. In addition, schizophrenia has also been reported to be associated with activation of inflammation and cell-mediated pathways [68] and those diagnosed with PTSD are reported to have higher levels of pro-inflammatory cytokines [69–72].

The relationship between inflammation, immune and endocrine changes and psychiatric morbidity is complex, and establishing temporality is challenging [66, 73]. A prospective community-based study of children (*n* = 1420) aged 9, 11 and 13 years with follow-up to 21 years examined bi-directional associations between the inflammation biomarker C-reactive protein (CRP) and depression [74]. This study found depression to be associated with later CRP levels, the relationship was stronger for cumulative episodes of depression, and the association persisted after controlling for obesity, smoking and medication use. In response to these results, Dantzer [73] postulated that the inflammatory status underlying elevated CRP levels at an early age could also influence the development or recurrence of depressive disorders in later life. As such, it may be worth considering the possibility that persistent inflammatory and immune changes triggered by burns may contribute in part to the psychiatric morbidity observed in this paediatric burn cohort.

### Study strength and limitations

This population-based cohort study examined long-term MH outcomes of paediatric patients with unintentional burns, had extensive follow-up time and a comparison uninjured group. Population-based linked health data reduces issues related to selection and losses to follow-up. While health administrative data do not routinely include clinical data, analyses were adjusted for socio-demographic factors and comorbidities. The socio-economic disadvantage variable used in this study represents a robust proxy-measure for a range of social behaviours derived and is correlated with lifestyle risk factors (e.g. diet, physical activity, smoking, alcohol) [28, 29]; however, a level of residual confounding may exist. In Australia, each person has access to medical and public hospital services and subsidised primary health care, regardless of socio-economic status. However, globally, burns occur disproportionately among racial and ethnic minorities and those of lower socioeconomic status [75]. Where access to such health care delivery is absent, these sectors of the community may be at higher risk of unmet long-term physical and MH care needs. Mandatory reporting laws exist in Australia to protect children from abuse, and the accuracy (86–95%) of ICD coding of intent-of-injury in hospital records is high [76]. MH hospitalisations represent more serious cases, and results of this study may underestimate the psychopathology experienced by children in the community. We anticipate that these results are generalisable to other countries of similar health delivery systems and demographics.

## Conclusions

The study highlights the need for early detection and management of symptoms of psychopathology after paediatric unintentional burns to prevent development/progression of MH disorders and possible detrimental effects on academic achievement and psychosocial wellbeing. These findings have implications for clinical management of paediatric burns during the initial admission, out-patient follow-up and longer-term MH surveillance via primary care. Future research focussing on underlying causes of these adverse trajectories will facilitate the development of targeted interventions aimed at prevention rather than treatment.

## Abbreviations

%TBSA: Percentage of total body surface area
95% CI: 95% confidence interval
ICD: International Classification for Diseases
IQR: Interquartile range
IRR: Incidence rate ratio
LOS: Length of stay
MH: Mental health
MHIS: Mental Health Information System
PY: Person-years
WAPBIP: Western Australian Population-based Burn Injury Project
